# Epidemiology of Brucellosis in Small Ruminants of Rural and Peri-Urban Areas of Multan, Pakistan

**DOI:** 10.1155/2024/8898827

**Published:** 2024-02-12

**Authors:** Mian Muhammad Awais, Bakhtawar Khadim, Masood Akhtar, Muhammad Irfan Anwar, Gohar Khadim, Abdul Sammad Ali Khan Shirwany, Halil Selcuk Biricik, Abdul Razzaq, Muhammad Sibtain Bhatti

**Affiliations:** ^1^One Health Research Laboratory, Department of Pathobiology, Faculty of Veterinary Sciences, Bahauddin Zakariya University, Multan, Pakistan; ^2^Veterinary Faculty, Afyon Kocatepe University, Afyonkarahisar, Türkiye; ^3^Animal Sciences Division, Pakistan Agricultural Research Council, Islamabad, Pakistan; ^4^Livestock and Dairy Development Department, Directorate of Multan Division, Multan, Pakistan

## Abstract

Brucellosis is a widespread zoonotic disease of veterinary and public health importance with considerably higher prevalence in developing/underdeveloped countries. This study reports the prevalence and risk determinants of brucellosis in small ruminants of peri-urban and rural areas of district Multan, Southern Punjab, Pakistan. For this purpose, sera samples (*n* = 392) of small ruminants were collected and subjected to preliminary screening using commercially available RBPT reagents followed by serodetection of brucellosis using multispecies i-ELISA kit (ID.vet, France). All the ELISA positive samples were confirmed by PCR using genus-specific primers, and frequencies of *Brucella* species in positive samples were enumerated using species-specific primers. Results indicated seropositivity rates of 9.69, 9.95, and 10.20% in study population using RBPT reagents of IDEXX-USA, ID.Vet-France, and VRI-Pakistan, respectively, with a statistically nonsignificant difference (*p* > 0.05). Results of ELISA showed an overall seroprevalence rate of 7.14% in target population with a slightly higher rate in sheep (7.65%) as compared to goat (6.63%) population (*p* = 0.695; OR = 1.16, 95% CI = 0.53, 2.57). Results revealed that out of total positive samples, *B. abortus* was detected in 60.71% of seropositive samples and *B. melitensis* was detected in 14.28% of positive samples. It was revealed that risk factors including body condition scores, hygienic conditions of the housing facility, farming system, reproductive disorders, educational status of farmers, and awareness of farmers about brucellosis had significant association with brucellosis in small ruminants of study area (*p* < 0.05). Conversely, farm/herd size, locality, gender, age, weight, and parity showed a nonsignificant association (*p* > 0.05) with brucellosis. In conclusion, brucellosis is prevalent in small ruminants of Multan, Pakistan. It is recommended to devise and implement effective control strategies with a major focus on raising awareness about brucellosis in farmers for the containment of infection in the region.

## 1. Introduction

Brucellosis is a widespread zoonotic disease of public health concern in most parts of the world including Pakistan [1]. It strikes both the animal and human populations throughout the world and has been ranked as second most important transmissible zoonotic disease by the World Health Organization with occurrence of more than 0.5 million cases per annum [2–4]. It inflicts huge economic losses in the livestock industry in terms of poor production performance, reproductive wastage, medication, and veterinary costs in addition to ban on the trade of susceptible food animals and their products [5, 6]. In Pakistan, the exact economic losses associated with brucellosis are not known but in our neighbor country (India) with similar geographical conditions and animal husbandry practices, it causes USD 3.4 billion losses in livestock industry in addition to USD 9.06 million losses associated with human brucellosis [7–9].

It is caused by Gram-negative facultative bacteria belonging to genus *Brucella* [10]. This genus has almost 20 different types of species with varying host preferences which affect nearly all domestic and wild mammalian species [11, 12]. In small ruminants, disease is characterized by abortion in third trimester, birth of immature kids/lambs, retention of fetal membranes, and stillbirth in female animals, whereas in males, it is manifested in the form of orchitis and infertility [13]. In endemic regions, cross-transmission of disease between humans and different animal species such as cattle, sheep, goats, camels, and pigs takes place by direct contact with blood, uterine discharge, and placenta of infected animals or by eating infected raw animal products, particularly unpasteurized milk and cheese [14]. In human beings, *B. melitensis* may cause severe infection that is usually characterized by fatigue, undulant fever, malaise, and osteomyelitis [15, 16]. Literature reveals that distribution of zoonotic *Brucella* species and their biovars vary with different geographical regions and husbandry practices. For instance, *B. abortus* is worldwide in distribution whereas *B*. *melitensis* and *B*. *suis* have irregular distribution patterns [17, 18]. The disease is more prevalent in countries with poor healthcare systems and inappropriate disease control programs [19, 20].

Previous studies revealed varied prevalence rates of brucellosis in small ruminants ranging from 0.70 to 41.90% [21, 22]. The prevalence of brucellosis is mainly influenced by different risk factors including age, sex, breed, reproductive status, and husbandry practices which determine the susceptibility of animals to the disease [23, 24].

The higher prevalence rates of brucellosis in animals pose a significant risk of disease transmission to workers of livestock farms, veterinary professionals, and slaughter-house personnel [25, 26]. In affected humans, the complicated disease manifestations, limited diagnostic facilities, recurrence of febrile episodes, and prolonged treatment make it difficult to treat the infection [27]. Keeping in view, it is of paramount importance to control brucellosis in animals by implementation of effective control programs including vaccination to avoid/minimize the public health concerns associated with this disease [5]. The early and accurate diagnosis followed by culling of affected animals might also play a significant role in reducing the burden of disease both in animals and human populations [28].

The culturing of *Brucella* in laboratory is considered as the “gold standard” method to identify the *Brucella* species [29, 30]. But it had always been challenging in most parts of the world to culture the bacteria which needs advanced biosafety requirements and good training to avoid intentional/unintentional biosecurity breach [31, 32]. Under the circumstances, serodiagnostic tests including milk ring test, Rose Bengal Plate Test (RBPT), serum agglutination test, Enzyme-Linked Immunosorbent Assay (ELISA), and complement fixation test are the most frequently used diagnostic tools which are relatively safer but each with variable sensitivity and specificity. However, molecular/PCR-based tests have also been developed in most industrialized countries for the detection of this multispecies illness with a high sensitivity and specificity [33–35]. An effective disease surveillance mechanism should be devised to determine the load of disease in livestock and human populations at regional and national levels for its efficient control. In this regard, limited data are available on the prevalence of brucellosis in small ruminants in few regions of Pakistan [10, 36], but data regarding the prevalence and associated risk factors of brucellosis in Southern Punjab containing a huge population of small ruminants are scarce. Keeping in view, this study aimed to determine the prevalence and associated risk factors of brucellosis in small ruminants of district Multan, Southern Punjab, Pakistan. Findings of this study will help to devise effective control measures to prevent brucellosis in small ruminants in the region.

## 2. Materials and Methods

### 2.1. Sampling Area and Target Population

**Figure 1:**
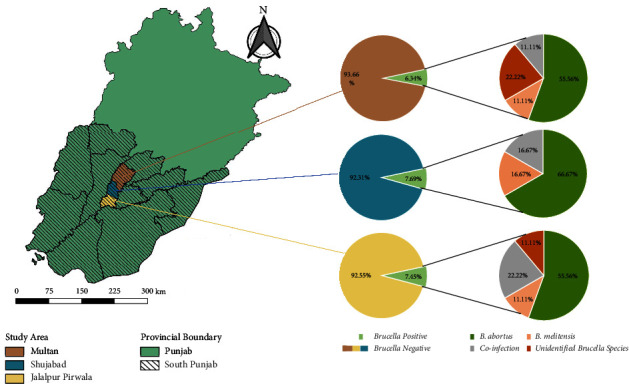
Map of study area showing tehsil-wise prevalence of brucellosis in small ruminants of district Multan, Pakistan.

The study was conducted in small ruminants (sheep and goat) population located in peri-urban and rural parts of Multan district of Southern Punjab, Pakistan, which is located on the Chenab River's bank. It is located at an elevation of 215 meters (740 feet) above sea level, between 30°11′44″ N and 71°28′31″ E (Figure 1). With a total area of 3,721 km^2^, it is one of the largest districts in Southern Punjab, Pakistan. Multan city, Multan Saddar, Jalalpur Pirwala, and Shujabad are the four tehsils that make up the district. It is one of the most populated districts in Punjab province, Pakistan. People in peri-urban and rural areas depend mainly on agriculture and animal production for their living. In terms of livestock, people rear large and small ruminants (cattle, buffalo, sheep, and goats), rural and commercial poultry, and camels for income in both domestic and commercial setups. It has extreme climatic conditions with maximum temperature in the summer (50°C) and minimum in the winter season (1°C) [37].

### 2.2. Study Period

The study was conducted from October 2020 to September 2021.

### 2.3. Determination of Size of Sample

The sample size was calculated by using the stratified sampling technique. The target population was divided into two strata including sheep and goats and sample size of each stratum was calculated by using the formula as follows:

(1) $$\begin{array}{c} n=\frac{Z^{2}\times P\left(1-P\right)}{\varepsilon^{2}}, \end{array}$$

where *n* = no. of samples; *Z* = 1.96 (95% level of confidence); *ε* = level of precision (5%); and *P* = expected prevalence (15%).

By using the abovementioned formula, a total of 392 blood samples were collected with a sample size of 196 from each of sheep and goat populations of study area.

### 2.4. Blood Sampling and Collection of Descriptive Epidemiological Data

Livestock farmers in peri-urban and rural regions of district Multan were mapped and approached for the collection of samples with the help of the Livestock and Dairy Development Department, Directorate of Multan Division, Government of Punjab. Blood sample (5 mL) was obtained aseptically from each animal's jugular vein and 3 mL blood was transferred into gel clot activator vacutainers to separate serum while 2 mL was transferred into EDTA-coated vacutainers to separate DNA from positive animals. All the samples were transferred to One Health Research Laboratory, Department of Pathobiology, Faculty of Veterinary Sciences, Bahauddin Zakariya University (BZU), Multan, Pakistan, for serological and molecular detection of brucellosis. The descriptive epidemiological data regarding possible risk factors including age, breed, gender (male/female), weight, abortion history, repeat breeding, retained fetal membranes, body condition score, hygienic conditions, farming pattern, educational status of farmers, and flock/herd size were collected on well-designed questionnaire.

### 2.5. Ethical Considerations

The study was approved from the Institutional Animal Welfare and Ethics Committee (No. FVS/AWEC-004/2020) followed by the Board of Studies of Department of Pathobiology, Faculty of Veterinary Sciences (FVS) and Advanced Studies and Research Board of Bahauddin Zakariya University, Multan, Pakistan (vide no. Acad/M.Phil/FVS/1535) for ethical issues pertaining to the humane sampling from small ruminants. Prior informed consent was obtained from all livestock farmers for using the data generated from the analysis of blood/sera samples of their animals for academic, research, and publication purposes without showing their identities.

### 2.6. Preliminary Screening of Brucellosis

All sera samples were screened for brucellosis using three different commercially available RBPT reagents, namely, RBPT antigen (VRI, Lahore, Pakistan), Pourquier Rose Bengale Ag (IDEXX, USA; Cat. #P00215), and Rose Bengal reagent (ID.vet®, France; Cat. #RSA-RB), according to the instructions of respective manufacturers. In brief, for each RBPT reagent, 30 *μ*L of test serum was mixed with an equal volume of *Brucella* antigen followed by shaking for 2–4 minutes. The formation of clear agglutination zone (approx. 2 cm diameter) on mixing reagent and test serum was considered a positive endpoint, whereas the sera with no agglutination were considered negative. For each reagent, test validation was done by using known positive and negative controls as a reference.

### 2.7. Serological Detection of Brucellosis Using Indirect Multispecies ELISA Kit

For serodetection of brucellosis, all the samples were subjected to ELISA using commercially available kit (ID-Screen Brucellosis Serum Indirect Multispecies, ID.vet®, France; product ref # BRUS-MS-10P). The test was performed according to the manufacturer's instructions.

### 2.8. Molecular Detection of *Brucella* Species by Polymerase Chain Reaction

All the ELISA positive samples were subjected to PCR using genus and species-specific primers to determine the frequencies of *Brucella* species in seropositive samples.

The primers targeting DNA sequence of *bcsp31* gene (Forward: 5′GCTCGGTTGCCAATATCAATGC3′ and Reverse: 3′GGGTAAAGCGTCGCCAGAAG5′ with product size of 151 bp) were used for the detection of *Brucella* spp. The species-specific primers targeting *alkB* gene (Forward: 5′GCGGCTTTTCTATCACGGTATTC3′ and Reverse: 3′CATGCGCTATGATCTGGTTACG5′ with product size of 136 bp) and BMEI1162 gene (Forward: 5′AACAAGCGGCACCCCTAAAA3′ and Reverse: 3′CATGCGCTATGATCTGGTTACG5′ with product size of 279pb) were used for the detection of *B*. *abortus* and *B*. *melitensis*, respectively [38]. In brief, genomic DNA of *Brucella* was extracted from blood of seropositive animals using genomic DNA-purification kit (GeneJET, Thermo Fisher Scientific; Catalog #K0721). The amplification of target genes was done using a thermal cycler (MultiGene Optimax, Labnet International, USA). The thermocycling conditions were as follows: initial denaturation for 10 min at 94°C, followed by 35 cycles of 94°C for 1 min, annealing at 57°C for 30 sec and 72°C for 1 min, and final extension/elongation for 5 min at 72°C. Then, this PCR product was further subjected to gel electrophoresis with 1.8% gel (agarose gel) as described by Lee et al. [39]. Then visualization of bands was done with UV Trans-illuminator (MS Major Science, USA) to determine the band size of PCR product(s) compared with DNA ladder (100 bp).

### 2.9. Statistical Analysis

The data collected from RBPT and ELISA results were subjected to statistical analysis using chi-square, odd's ratio, and confidence intervals (95%) using Minitab Ver.17.0 and R (version 4.2.0) using RStudio (version 2022.02.3 + 492) as an interface. The inter-rater reliability of serological tests was determined by using Cohen's kappa statistic. The differences between different variables were considered significant at *p* < 0.05.

## 3. Results

### 3.1. Screening and Comparison of Brucellosis Using Different Commercially Available RBPT Antigens

Results revealed that maximum seropositivity was recorded with RBPT-VRI (10.20%) followed by RBPT-ID.vet (9.95%) and RBPT-IDEXX (9.69%), whereas the difference was nonsignificant statistically (*p* = 0.972). It indicated that all the antigens are equally suitable for preliminary screening of brucellosis in small ruminants. A similar trend was observed in species-wise analysis of small ruminants for screening of brucellosis using RBPT antigens from different manufacturers (Table 1).

### 3.2. Overall and Species-Wise Prevalence of Brucellosis Using Multispecies i-ELISA Kit

The overall seroprevalence of brucellosis using multispecies i-ELISA Kit was 7.14% (*n* = 28/392; 95% CI = 4.87–10.12%). Results also showed comparatively higher prevalence rate in sheep (7.65%; 15/196) as compared to goat (6.63%; 13/196), whereas the difference was nonsignificant (*p*=0.695; OR = 1.16; 95% CI = 0.53–2.57) (Table 2). The kappa statistics showed an almost perfect agreement between different RBPT-based tests (kappa value between 0.81 and 1.00), whereas ELISA had substantial agreement with different RBPT-based tests (kappa value between 0.61 and 0.80) (Table 3).

### 3.3. Molecular Detection of *Brucella* Species in ELISA Positive Samples Using Polymerase Chain Reaction

The PCR analysis confirmed the presence of *Brucella* in all the ELISA positive samples using genus-specific primers. On the other hand, the results of PCR using species-specific primers revealed that out of total positive samples, *B. abortus* was detected in 60.71% (*n* = 17/28) and *B. melitensis* was detected in 14.28% (*n* = 4/28) of positive samples. The coprevalence of *B. abortus* and *B. melitensis* in positive samples was 14.28% (*n* = 04/28). The tehsil-wise prevalence and distribution of *Brucella* species in target population are shown in Figure 1.

### 3.4. Farm/Flock Level Prevalence of Brucellosis in Small Ruminants

**Figure 2:**
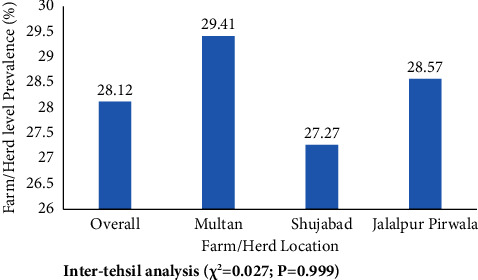
Farm/herd level prevalence of brucellosis in small ruminants.

A total of 64 sheep and goat flocks located in peri-urban and rural areas of different tehsils of district Multan were targeted in this study. Results revealed that animals at 18 flocks were positive for brucellosis indicating its flock level prevalence of 28.12%. In tehsil-wise data analysis, it was revealed that flock level prevalence was highest in tehsil Multan (29.41%) followed by those of Shujabad (27.27%) and Jalalpur Pirwala (28.57%); however, the difference was statistically nonsignificant (*p*=0.999) (Figure 2).

### 3.5. Association of Sociodemographic Factors with the Prevalence of Brucellosis in Small Ruminants

The results regarding the association of sociodemographic factors with the prevalence of brucellosis in small ruminants of study area are shown in Table 4. No association was found between age groups and overall/species-wise prevalence of brucellosis in small ruminants (*p* > 0.05). Similarly, no significant association of brucellosis was observed with herd size, gender, and breed of small ruminants (*p* > 0.05). Body condition scores ranging from 1 (very thin) to 5 (very fat) were used as a criterion to assess the muscle and fat deposition in small ruminants. Scoring was done based on feeling around the vertebral column in the loin region. The animals with BCS 1-2 were considered poor, with 3 as medium and above 3 as good. Results revealed a significant association (*χ*^2^ = 9.751) of poor body score with prevalence of brucellosis in overall small ruminant (*p* = 0.008), sheep (*p* = 0.030), and goat (*p* = 0.038) populations. In female population, data were divided into 3 groups with respect to no. of parities, namely, (i) with ≤2 parities, (ii) 3 to 4 parities, and (iii) >4 parities. The data analysis revealed that there is no association between no. of parities and prevalence of brucellosis (*p* = 0.757). With respect to physiological status of females, the pregnant females showed slightly higher prevalence of brucellosis (8.33%) as compared to nonpregnant (6.21%) although the difference was statistically nonsignificant (*p* = 0.543). The small ruminants raised at farms with good hygienic housing conditions showed lesser prevalence rate (3.27%) as compared to those raised under poor hygienic conditions (11.80%), and the difference was statistically significant (*χ*^2^ = 10.652; *p* = 0.001). It was also revealed that small ruminants raised under poor hygienic conditions had 3.885 (95% CI = 1.675–10.20) times higher odds of brucellosis as compared to animals raised in good hygienic housing conditions. Similarly, results showed a significant association between farming system and prevalence of brucellosis (*χ*^2^ = 5.026; *p* = 0.025). The animals raised in mixed farming system with large ruminants showed higher prevalence rate as compared to those raised at exclusive small ruminant farms/housing facilities. The odds of brucellosis was 0.397 (95% CI = 0.159–0.898) times lower in small ruminants raised in exclusive small ruminant farming system as compared to those raised in mixed farming system.

### 3.6. Association of Reproductive Disorders with Prevalence of Brucellosis in Small Ruminants

Results showed a significant association of reproductive disorders with prevalence of brucellosis in small ruminants (Table 5). The animals with abortion history showed higher prevalence rate (25.86%) as compared to those without any abortion history (2.92%) indicating a significant association between abortion history and prevalence of brucellosis in small ruminants in the study area (*p*=0.000). Results showed that animals with repeat breeding history (RBH) showed higher prevalence rate (17.19%) as compared to those with no RBH (4.30%) and the difference was statistically significant (*p*=0.000). Similarly, the animals with RFM showed higher prevalence rate (16.36%) as compared to those with no RFM history (4.82%), and statistical analysis revealed that the difference is significant (*p*=0.001).

### 3.7. Association of Educational Status and Awareness Level of Farmers with Prevalence of Brucellosis in Small Ruminants

With respect to the educational status of farmers, data were analyzed into two groups, namely, (i) educated and (ii) noneducated farmers. Data analysis revealed that small ruminants raised by farmers having no education showed significantly higher prevalence (14.92%) as compared to those of educated farmers (*p*=0.000). Similarly, it was also revealed that lack of awareness of farmers about brucellosis had significant association with prevalence of brucellosis in small ruminants (*p*=0.036) (Table 6).

## 4. Discussion

Brucellosis is a zoonotic disease that is highly contagious and results in several adverse outcomes. In rare cases, it may also cause arthritis in small ruminants [41]. It has worldwide distribution and is endemic in many developing/underdeveloped countries including Pakistan [9, 42]. In most of the developing countries like Pakistan, the small ruminants and their products play an important role in the national economy and also support millions of people living in rural and urban areas as the source of meat, milk, cheese, cream, yogurt, hooves, horns, and leather [43]. Literature revealed that distributions of *Brucella* species vary with different geographical regions and husbandry practices [44]. Due to wide variation in prevalence and associated risk factors of brucellosis, this study was conducted to determine the prevalence and associated risk factors of brucellosis in small ruminants of peri-urban and rural areas of district Multan, Pakistan.

In this study, the overall seropositivity rates with RBPT reagents were 9.69% with IDEXX-USA, 9.95% with ID.vet-France, and 10.20% with VRI-Pakistan with a nonsignificant difference in findings obtained with different RBPT reagents (*p* > 0.05). However, the locally manufactured RBPT reagent of VRI-Pakistan was advantageous to use for being cheapest among all the RBPT reagents used in this study. The results of sero-ELISA of all the collected samples revealed an overall seroprevalence of 7.14% in small ruminants which were further confirmed by PCR using genus- and species-specific primers. All the seropositive samples were positive for the genome of genus *Brucella*; however, the genome of *B. abortus* was detected in 60.71% (17/28) and *B. melitensis* was detected in 14.28% of *Brucella* positive samples and coprevalence of both species was also found to be 14.28%. It was also revealed that difference in prevalence of brucellosis was nonsignificant between sheep and goat populations. Similar to our findings, Shome *et al.* [9] also reported an apparent prevalence of 7.45% in small ruminants of our neighboring country, India, but contrary to our findings, they recorded significantly higher prevalence in sheep as compared to goats (*p* < 0.0001). In contrast to our findings, some previous studies showed lower prevalence rates of small ruminant brucellosis such as 1.79% in Tselemti district, Northern Ethiopia [45], 1.8% in Debre Zeit and Modjo export abattoirs of Oromia Regional State of Ethiopia [46], 1.2% in Gambia [47], 5.1% in different farms of Punjab, Pakistan [36], 6.26% in Southeast Europe [48], 0.4% in Northwest Ethiopia [49], 3.4% in Quetta and its surrounding areas of Pakistan [50], 3.2% in Borena, Southern Ethiopia [51], and 4.5% in China [52]. On the other hand, as compared to our findings, higher prevalence rates had also been reported in some parts of the world including South Omo Zone, Ethiopia [53], Iraq [54], India [23], Thailand [55], Nigeria [56], and Jordan [57]. The varied prevalence rates of brucellosis in different regions might be due to differences in sensitivities and specificities of techniques used for diagnosis of bacteria, husbandry practices, and geoclimatic conditions which might be more suitable (for bacteria to grow) in regions with higher prevalence rates [58].

The flock/herd level prevalence of brucellosis in small ruminants was 28.12% with nonsignificant difference in flock level prevalence rates at tehsil levels (*p*=0.99) although highest prevalence was recorded in tehsil Multan (29.41). Any flock containing at least one positive animal for *Brucella* antibodies was considered as positive. Previously, Gompo et al. [59] reported flock level prevalence rates of 30 and 3.33% for brucellosis in sheep and goat populations of Southern Nepal, respectively. Sharifi et al. [60] also reported a prevalence rate of 21% for brucellosis in small ruminants of Southeastern Iran. The higher flock level prevalence might be correlated with the differences in husbandry practices, poor biosecurity, and more chances of cross-transmission of infection in animals kept in closed confinement in small ruminant flocks raised in study area in addition to intermixing of different flocks in the same grazing areas [61, 62].

The analysis regarding prevalence of brucellosis in different tehsils of Multan showed a nonsignificant difference between the tehsils (*p*=0.895). Similar to our findings, Geletu et al. [13] also reported a nonsignificant difference in small ruminant brucellosis in different districts of West Hararghe Zone of Oromia Regional State, Eastern Ethiopia (*p*=0.438). Saeed et al. [44] also reported varied prevalence rates of brucellosis in different regions of Central Punjab, Pakistan, but no statistical difference had been reported in this study. Previous literature revealed that geoclimatic conditions might influence the prevalence of various diseases in different geographical regions [58, 63]; however, nonsignificant association in our study might be speculated due to similar geoclimatic conditions in all regions (tehsils) of our study area.

In our study, gender-wise analysis revealed apparently higher prevalence rate of brucellosis in males of small ruminants as compared to females; however, difference was nonsignificant (*p* > 0.05). Similar to our findings, Nguna et al. [64]; Ogugua et al. [65]; and Adugna et al. [2] also reported a nonsignificant association between sex and caprine brucellosis. However, on the other hand, Sorsa et al. [66]; Tschopp et al. [67]; and Addis and Desalegn [68] showed a significant association of sex with ovine brucellosis and reported significantly higher prevalence rate in ewes as compared to rams. Variation in sex-wise prevalence of brucellosis in different studies might be due to differences in geographical locations, age groups, sampling techniques, and sample sizes. In our study area, percent population of males in herds of small ruminants is lesser as compared to females because males are being sold at young age for slaughtering/meat. Due to the abovesaid reason, the sample size of males was relatively smaller in this study which might be considered as a limitation of this study.

The difference in age-wise prevalence of brucellosis was found statistically nonsignificant; however, prevalence rates were apparently higher in adult sheep and goats aged >3 years. Our findings are consistent with those of Tschopp et al. [67] and Ashagrie et al. [69] who also reported a nonsignificant association of age with prevalence of brucellosis in small ruminants. Conversely, Edao et al. [51] reported a significant association of age groups with *Brucella* seropositivity in sheep and goat population of Borena, Southern Ethiopia, but similar to our findings, they also reported that small ruminants aged >3 years were more likely to be seropositive which might be due to increased chance of infection with increasing age. Furthermore, seropositivity to *Brucella* might also increase with age due to prolonged duration of humoral responses in affected animals along with continued exposure to pathogen especially in herds where animals are kept over a longer period of time [70].

The difference in breed-wise prevalence of brucellosis was nonsignificant both in sheep and goat populations. Similar to our findings, Ullah et al. [36] and Olufemi et al. [56] also reported a nonsignificant correlation between breed and prevalence of brucellosis in small ruminants of Pakistan and Nigeria, respectively. However, Dogo et al. [71] and Natesan et al. [22] also reported a significant correlation of breeds of small ruminants with prevalence of brucellosis in small ruminants of Nigeria and India, respectively. The variations in breed-wise prevalence of brucellosis in small ruminants of different regions might be due to differences in management practices and immunogenic response of different breeds to resist against invading pathogens including brucellosis [22, 72, 73].

In our study, a nonsignificant association (*p* > 0.05) was recorded between live body weight of the small ruminants and prevalence of brucellosis. However, apparently higher prevalence rates were recorded in animals with poor body weights. No such correlation has been reported previously. However, it might be speculated that poor body weight might be due to malnourished status of animals leading to poor immunity [74, 75] and thus higher prevalence of different diseases including brucellosis in animals with poor body weight gains. On the other hand, brucellosis had also been well documented for causing weight loss in infected animals [76–78].

It was revealed that body condition scores were significantly associated (*p* < 0.05) with prevalence of brucellosis in small ruminants with highest prevalence in animals with poor BCS (very thin and thin). Previously, Tsegaye et al. [79]; Ullah et al. [36]; and Sorsa et al. [66] also reported a significant association of BCS with brucellosis in small ruminants. Similarly, Waktole et al. [80] also reported similar findings in camel brucellosis. Contrary to our findings, Robi and Gelalcha [81] reported a nonsignificant association of BCS with brucellosis in cattle. It might be speculated that poor BCS enhanced the susceptibility to other diseases when an animal is already infected with brucellosis, or the loss in BCS might be due to brucellosis itself.

A significant association was detected between farm hygiene and prevalence of brucellosis in small ruminants. The authors of [82, 83] also reported a positive correlation of farm hygiene and sanitary conditions with brucellosis in small ruminants. A possible reason could be higher exposure and susceptibility to infectious diseases including brucellosis in animals kept at farms with poor hygienic conditions.

Our results indicated significantly higher prevalence of brucellosis in small ruminants kept with other *Brucella-*susceptible animals (large ruminants). A possible reason behind higher prevalence of brucellosis in mixed farming system might be correlated with its ability of cross-transmission among various susceptible species of large and small ruminants [25, 84].

In this study, a nonsignificant association (*p* > 0.05) was detected between flock/herd size and brucellosis in small ruminants. Contrary to our findings, Sorsa et al. [66]; Gompo et al. [59]; and Jamil et al. [10] reported a significant association between flock size and brucellosis in small ruminants of Ethiopia, Nepal, and western border areas of Pakistan, respectively. However, similar to our findings, Al-Majali et al. [85] also reported a nonsignificant association between flock size and brucellosis in Awassi sheep in Jordan. The varied results in different studies might be due to inconsistent production systems and husbandry practices in different parts of the world.

Reproductive losses in terms of abortion, infertility, delivery of weak offspring, sterility, and stillbirth are common signs of brucellosis in small ruminants [86]. Majority of cases of abortion in ruminants are attributed to *Brucella* infection [51, 87, 88]. Similarly, in our study, a significant association (*p* < 0.05) was detected between prevalence of brucellosis and reproductive disorders including abortion history, repeat breeding, and retained fetal membranes in small ruminants. Similarly, Ullah et al. [36] also reported a positive correlation between reproductive disorders and brucellosis in small ruminants kept at Institutional Livestock Farms in Punjab, Pakistan. Furthermore, Edao et al. [51]; Tsegaye et al. [79]; and Matope et al. [89] also reported a significant association of abortion history with small ruminants' brucellosis in different parts of the world. Addis and Desalegn [68] and Kelkay et al. [45] also reported significant association of ovine brucellosis with history of both abortion and retained fetal membranes in Ethiopia. Ibrahim and Zaghawa [90] also reported a higher prevalence rate of brucellosis in small ruminants (33%) as compared to those without repeat breeding history (15.5%) although statistical significance was not described. Contrary to our findings, Nguna et al. [64] reported a nonsignificant association of abortion history with brucellosis in cattle and goats in Uganda. In our study area, higher prevalence rates in animals with reproductive disorders might be correlated with behavior of farmers such as poor hygiene at farms, lack of culling the infected animal heads, purchase and mixing of new animals in existing flocks without appropriate screening and quarantine, and lack of awareness about brucellosis. This is a threatening situation apprehending the zoonotic implications of brucellosis as an occupational health hazard for small ruminant holders.

Our study showed a nonsignificant difference in prevalence rates of brucellosis in pregnant and nonpregnant females of small ruminants. Our results are in agreement with findings of Kelkay et al. [45] and Uddin et al. [91] who also reported a nonsignificant association between pregnancy and brucellosis in small ruminants. In contrast, a significant correlation between pregnancy status and brucellosis in small ruminants had also been reported in Tellalak district of Afar region, Ethiopia [92].

In our study, no significant association was found between number of parities and brucellosis in small ruminants. However, an increasing prevalence rate was recorded with increase in number of parities. Previously, a significant association between prevalence of brucellosis and parity had also been reported in some previous studies [59, 66, 93]. It might be speculated that age of animals increases with increasing number of parities and attributes to increased chance of infection with increasing age [70].

A significant association of educational status of farmers and their awareness level about brucellosis was found with prevalence of brucellosis in small ruminants of district Multan, Pakistan. Several previous studies reported that educational status is significantly associated with awareness and knowledge about different diseases including brucellosis leading to lower prevalence rates of brucellosis in communities with better educational status and awareness about the disease [94–99]. It is speculated that farmers with better educational status can improve farm management practices and maintain better hygienic conditions to prevent or reduce the chances of disease transmission and spread [100]. Keeping in view, an awareness campaign should be planned by veterinary and public health agencies regarding transmission and control of brucellosis.

## 5. Conclusions and Recommendations

In conclusion, brucellosis is endemic in small ruminants of district Multan, Pakistan, with an overall prevalence rate of 7.14%. Prevalence of brucellosis in small ruminants was significantly associated with body condition scores, hygienic conditions of the farm, farming system, abortion history, retained fetal membranes, repeat breeding, educational status of farmers, and awareness of farmers about brucellosis. On the other hand, some other factors including flock/herd size, locality, gender, age, and parity showed a nonsignificant association with brucellosis in small ruminants of district Multan, Pakistan. It is recommended to devise and implement effective control strategies with a major focus on inculcating awareness about brucellosis in small ruminant farmers for the containment of infection in the region.

## Figures and Tables

**Table 1:** Screening and comparison of brucellosis using different commercially available RBPT antigens.

	% seropositivity with RBPT (*n*/*T*) (95% CI prevalence)			*p* value	*χ* ^2^
	VRI	IDEXX	ID.vet		
Overall	10.20 (40/392) (7.42, 13.57)	9.69 (38/392) (7.04, 13.05)	9.95 (39/392) (7.21, 13.31)	0.972	0.057
Sheep	10.71 (21/196) (6.96, 15.91)	10.20 (20/196) (6.43, 15.16)	11.22 (22/196) (7.19, 16.43)	0.948	0.107
Goat	9.69 (19/196) (6.10, 14.63)	9.18 (18/196) (5.67, 14.11)	8.67 (17/196) (5.13, 16.36)	0.941	0.122

RBPT, Rose Bengal Plate Test.

**Table 2:** Overall and tehsil-wise prevalence of brucellosis using multispecies i-ELISA kit.

Small ruminant	Total samples (*n*)	Positive samples (*n*)	Seroprevalence (95% CI)	*p* value	*χ* ^2^	OR	OR (95% CI)	
							Lower limit	Upper limit
Overall	392	28	7.14 (4.87, 10.12)	—	—	—		

Sheep	196	15	7.65 (4.37, 12.11)	0.695	0.154	1.16	0.53	2.57
Goat	196	13	6.63 (3.60, 11.04)					

**Table 3:** Strength of agreement between different serodiagnostic tests of brucellosis using kappa statistics.

	RBPT-VRI	RBPT-IDEXX	RBPT-ID.vet	ELISA
RBPT-VRI	—	0.86	0.87	0.74
RBPT-IDEX	0.86	—	0.87	0.74
RBPT-IDVET	0.87	0.87	—	0.72
ELISA	0.74	0.74	0.72	—

*Note*. Kappa values ≤0 = no agreement; 0.01–0.20 = none to slight agreement; 0.21–0.40 = weak agreement; 0.41–0.60 = moderate agreement; 0.61–0.80 = substantial agreement; 0.81–1.00 = almost perfect agreement [40].

**Table 4:** Association of sociodemographic factors with the prevalence of brucellosis in small ruminants of district Multan, Pakistan.

Demographic characters	Prevalence (*n*/*T*) (95% CI)	Chi-square (*χ*^2^)	*p* value	OR	OR (95% CI)	
					Lower limit	Upper limit
Age						
Overall						
≤3 years	5.18 (10/193) (2.65, 9.15)	2.205	0.138	0.554	0.238	1.122
>3 years	9.04 (18/199) (5.59, 13.91)					
Sheep						
≤3	3.70 (3/81) (1.01, 10.08)	3.047	0.081	0.344	0.073	1.145
>3 years	10.43 (12/115) (5.78, 17.16)					
Goat						
≤3	6.25 (7/112) (2.82, 12.21)	0.062	0.804	0.864	0.271	2.85
>3 years	7.14 (6/84) (2.19, 14.53)					
Gender						
Overall						
Female	6.56 (24/366) (4.25, 9.60)	2.852	0.091	0.377	0.130	1.407
Male	15.38 (4/26) (5.43, 34.09)					
Sheep						
Female	6.67 (12/180) (3.69, 11.21)	3.036	0.081	0.303	0.081	1.53
Male	18.75 (3/16) (5.31, 43.44)					
Goat						
Female	6.45 (12/186) (3.57, 10.85)		N/A			
Male	10.00 (1/10) (0.51, 44.45)					
Breed						
Sheep						
Thali	4.88 (4/82) (1.67, 11.79)		N/A			
Kajli	0.00 (0/6)					
Desi	0.00 (0/3)					
Kachi	0.00 (0/1)					
Lohi	4.76 (2/42) (0.85, 15.92)					
Non-descript	5.00 (4/80) (1.72, 12.08)					
Goat						
Beetal	6.94 (5/72) (2.77, 14.92)	0.613	0.736			
Nachhi	9.37 (3/32) (2.60, 24.31)					
Non-descript	5.43 (5/92) (2.16, 12.13)					
Body condition score						
Overall						
Poor	11.76 (14/119) (6.85, 18.68)	9.751	0.00			
Medium	9.01 (10/111) (4.61, 15.93)					
Good	2.47 (4/162) (0.85, 5.96)					
Sheep						
Poor	15.69 (8/51) (7.12, 28.07)	7.038	0.030			
Medium	6.67 (5/75) (2.66, 14.51)					
Good	2.86 (2/70) (0.51, 9.52)					
Goat						
Poor	8.82 (6/68) (3.91, 17.96)	6.541	0.038			
Medium	13.89 (5/36) (5.63, 28.68)					
Good	2.17 (2/92) (0.38, 7.24)					
Parity						
Overall						
≤2	5.55 (9/162) (2.80, 10.26)	0.557	0.757			
>2 but ≤4	6.98 (9/129) (3.54, 12.55)					
>4	8.00 (6/75) (3.53, 16.28)					
Sheep						
≤2	6.67 (4/60) (2.31, 16.14)	0.240	0.887			
>2 but ≤4	7.69 (5/65) (3.07, 16.51)					
>4	5.45 (3/55) (1.50, 14.88)					
Goat						
≤2	4.90 (5/102) (1.95, 10.93)	2.832	0.243			
>2 but ≤4	6.25 (4/64) (2.16, 15.13)					
>4	15.00 (3/20) (4.21, 36.94)					
Pregnancy						
Overall						
Yes	8.33 (5/60) (3.33, 17.88)	0.369	0.543	1.401	0.440	3.690
No	6.21 (19/306) (3.80, 9.52)					
Sheep						
Yes	10.00 (2/20) (1.8, 31.61)	0.402	0.526	1.75	0.231	7.511
No	6.25 (10/160) (3.20, 11.04)					
Goat						
Yes	7.50 (3/40) (2.07, 19.41)	0.093	0.761	1.271	0.258	4.612
No	6.16 (9/146) (3.12, 11.12)					
Flock size						
Overall						
Up to 20 animals	6.52 (6/92) (2.87, 13.27)	0.147	0.929			
From 21 to 50	7.81 (10/128) (4.00, 13.81)					
More than 50 animals	6.98 (12/172) (3.86, 11.73)					
Sheep						
Up to 20 animals	9.38 (3/32) (2.60, 24.31)	0.376	0.829			
From 21 to 50	8.62 (5/58) (3.45, 18.51)					
More than 50 animals	6.60 (7/106) (2.99, 12.90)					
Goat						
Up to 20 animals	5.00 (3/60) (1.37, 13.63)	0.382	0.826			
From 21 to 50	7.14 (5/70) (2.85, 15.31)					
More than 50 animals	7.57 (5/66) (3.02, 16.25)					
Housing conditions						
Good	3.27 (7/214) (1.48, 6.59)	10.652	0.001	3.885	1.675	10.20
Poor	11.80 (21/178) (7.67, 17.26)					
Farming system						
Small ruminants only	4.17 (8/192) (1.84, 7.88)	5.026	0.025	0.397	0.159	0.898
Mixed farming	10.00 (20/200) (6.31, 14.85)					

**Table 5:** Association of reproductive disorders with prevalence of brucellosis in small ruminants of district Multan, Pakistan.

Reproductive disorders	Prevalence (*n*/*T*) (95% CI)	Chi-square (*χ*^2^)	OR	OR (95% CI)		*p* value
				Lower limit	Upper limit	
Abortion history						
Overall						
Yes	25.86 (15/58) (15.92, 38.62)	41.919	11.381	4.732	28.950	0.000
No	2.92 (9/308) (1.45, 5.39)					
Sheep						
Yes	26.67 (8/30) (13.08, 44.86)	23.143	12.729	3.614	53.083	0.000
No	2.66 (4/150) (0.91, 6.44)					
Goat						
Yes	25.00 (7/28) (11.38, 44.47)	18.790	9.899	2.840	37.303	0.000
No	3.16 (5/158) (1.24, 7.05)					
Repeat breeding history						
Overall						
Yes	17.19 (11/64) (9.45, 28.59)	14.304	4.594	1.905	10.918	0.000
No	4.30 (13/302) (2.33, 7.17)					
Sheep						
Yes	17.24 (5/29) (7.04, 35.73)	6.213	4.266	1.145	14.836	0.013
No	4.63 (7/151) (2.09, 9.06)					
Goat						
Yes	17.14 (6/35) (7.73, 32.48)	8.165	4.937	1.414	17.294	0.004
No	3.97 (6/151) (1.74, 8.37)					
History of retention of fetal membranes						
Overall						
Yes	16.36 (9/55) (8.54, 28.73)	10.158	3.863	1.526	9.292	0.001
No	4.82 (15/311) (2.75, 7.76)					
Sheep						
Yes	18.52 (5/27) (7.59, 36.65)	7.171	4.711	1.258	16.492	0.007
No	4.57 (7/153) (2.06, 8.94)					
Goat						
Yes	14.29 (4/28) (5.02, 31.60)	3.352	3.150	0.762	11.100	0.067
No	5.06 (8/158) (2.24, 9.59)					

**Table 6:** Association of educational status and awareness level of farmers with prevalence of brucellosis in small ruminants of district Multan, Pakistan.

Groups	Total samples (*n*)	Positive samples (*n*)	Negative samples (*n*)	Prevalence (95% CI)	*p* value	*χ* ^2^	OR	OR (95% CI)	
								Lower limit	Upper limit
*Educational status of farmers*									
Educated (primary to master)	258	8	250	3.10 (1.37, 5.87)	0.000	18.592	0.185	0.074	0.421
Noneducated	134	20	114	14.92 (9.41, 21.54)					

*Awareness about brucellosis*									
Yes	142	5	137	3.52 (1.39, 7.84)	0.036	4.403	0.369	0.120	0.928
No	250	23	227	9.20 (6.05, 13.46)					

## Data Availability

Data will be made available on reasonable request to the corresponding author.

## Supplementary Materials

Supplementary Materials The gel images of PCR products of *Brucella* species can be seen in supplementary material.
