# Supplementary figures and images for: Epidemiology of Brucellosis in Small Ruminants of Rural and Peri-Urban Areas of Multan, Pakistan
Source: Can J Infect Dis Med Microbiol. 2024 Feb 12;2024:8898827. doi: 10.1155/2024/8898827 (PMC10881254; doi:10.1155/2024/8898827)

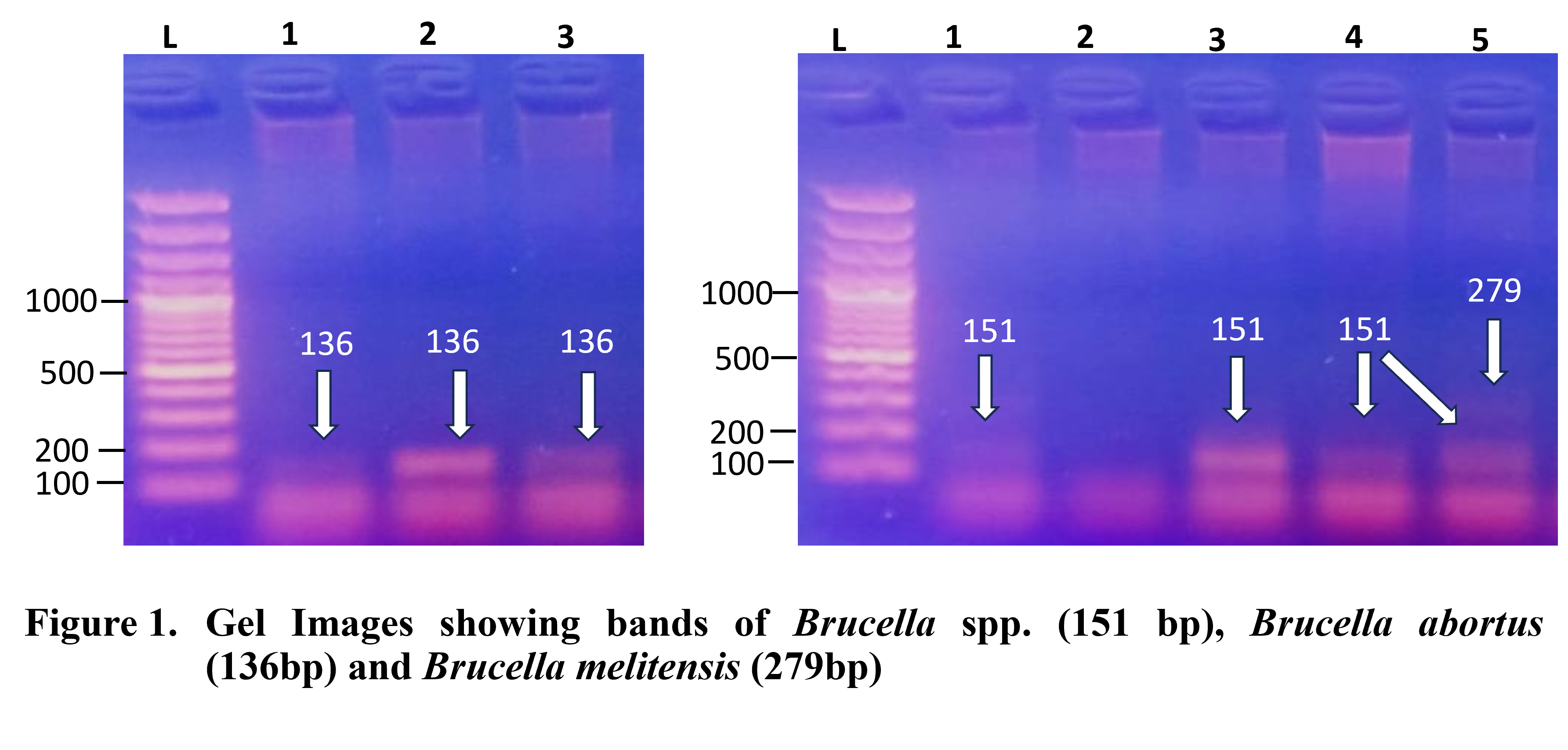

Supplement: Supplementary Materials — The gel images of PCR products of Brucella species can be seen in supplementary material. [file 8898827.f1.png]
